# The mediating effect of arteriosclerosis on the relationship between sarcopenia and cognitive impairment: a retrospective analysis

**DOI:** 10.3389/fmed.2026.1764510

**Published:** 2026-05-29

**Authors:** GangWei Zhang, YiJie Wang, DaoYuan Guo, Ci Lv, YuanYuan Du, XiaoMei Wang

**Affiliations:** 1 Department of General Practice of Jiangbei Campus, The First Affiliated Hospital of Army Medical University, Chongqing, China; 2 School of Medicine, Chongqing University, Chongqing, China; 3 Department of Geriatrics, The First Affiliated Hospital of Army Medical University (The Southwest Hospital of AMU), Chongqing, China

**Keywords:** arteriosclerosis, cognitive impairment, effect of mediation, healthy aging, sarcopenia

## Abstract

**Objective:**

Sarcopenia and arteriosclerosis are important factors affecting cognitive function, but their interrelationships remain unclear. This study investigated whether arteriosclerosis mediates the mediation effect between sarcopenia and cognitive impairment.

**Methods:**

A total of 105 patients aged ≥ 60 years from geriatric clinics were enrolled. Sarcopenia was diagnosed according to the 2019 Asian Working Group for Sarcopenia criteria. Cognitive function was assessed using the Mini-Mental State Examination (MMSE), and arterial stiffness was evaluated by brachial-ankle pulse wave velocity (baPWV). Multivariate linear regression was used to analyze associations, and mediation effects were tested with the bootstrap method.

**Results:**

99 patients met the inclusion criteria, among whom 34 had cognitive impairment. This group showed a higher prevalence of sarcopenia and significantly elevated baPWV. Regression analyses revealed negative associations between baPWV and MMSE (β = –0.004, *P* < 0.001), skeletal muscle mass index (SMI) (β = –201.239, *P* < 0.001), and grip strength (β = –13.732, *P* = 0.025). MMSE was positively associated with SMI (β = 1.267, *P* = 0.040) and grip strength (β = 0.260, *P* < 0.001). Mediation analysis indicated that baPWV partially mediated the associations of grip strength (13.9%) and SMI (31.8%) with MMSE, but these effects lost significance after adjustment for confounders.

**Conclusion:**

Sarcopenia is significantly associated with cognitive impairment and increased arteriosclerosis in elderly individuals. Although arteriosclerosis may be involved in this relationship, its mediating role is not robust after adjustment for confounding factors and remains inconclusive. Further longitudinal studies are required to clarify the underlying mechanisms.

## Introduction

Population aging has become a pressing global public health challenge, with the number of individuals aged ≥ 65 years increasing annually (1). The high prevalence of chronic conditions among older adults including sarcopenia, metabolic syndrome, malnutrition, and dementia imposes an increasing burden on healthcare systems.

Cognitive dysfunction is a hallmark of aging, characterized by marked decline or impairment in memory, executive function, judgment, learning, and other cognitive abilities (2). Sarcopenia is an age-related, progressive, and generalized skeletal muscle disorder defined by accelerated loss of muscle mass and function (3). Epidemiological studies indicate that the prevalence of sarcopenia in individuals aged ≥ 60 years ranges from 10 to 27%. Sarcopenia is strongly associated with adverse outcomes such as falls, frailty, and mortality (4). Arteriosclerosis, characterized by reduced vascular elasticity and increased arterial stiffness, is another age-related condition. Although these represent three distinct chronic progressive disorders, accumulating evidence suggests that they share underlying pathophysiological links, and many elderly individuals present with two or even all three simultaneously.

The interrelationship among sarcopenia, cognitive dysfunction, and arteriosclerosis has not been fully elucidated. A 3-year longitudinal study of 2,982 older adults (≥ 60 years) demonstrated that the prevalence of sarcopenia and probable sarcopenia increased from 8.5 to 29.6%, and both probable sarcopenia and sarcopenia were associated with an elevated risk of cognitive dysfunction compared with non-sarcopenia (5). Moreover, multiple cross-sectional studies have reported significant associations between sarcopenia and cognitive decline (6–8). Sarcopenia and arteriosclerosis share common risk factors, particularly age-related mechanisms. A multicenter study of community-dwelling older adults further confirmed that arteriosclerosis remained independently associated with sarcopenia after adjustment for multiple confounders (9). However, few studies have simultaneously examined the complex interrelationship among the three conditions, particularly the potential mediating role of arteriosclerosis in the association between sarcopenia and cognitive dysfunction.

Elucidating these relationships may provide important insights into the mechanisms underlying cognitive decline in the elderly and inform strategies for its prevention and management. Therefore, this study aimed to explore the associations among sarcopenia, arteriosclerosis, and cognitive dysfunction, with a particular focus on the mediating role of arteriosclerosis.

## Materials and methods

### Research participants

This study adopted a retrospective research design and collected patients who visited the Geriatrics Department of Southwest Hospital from October 2024 to January 2025 as the research subjects. Inclusion criteria: (1) Age > 60 years old; (2) Upon admission, the skeletal muscle mass index (SMI), grip strength, Mini-Mental State Examination Scale (MMSE) and brachial-ankle artery Pulse Wave Velocity (baPWV) examinations have been completed. Exclusion criteria: (1) Severe cardiovascular or cerebrovascular diseases or advanced tumors; (2) Those equipped with cardiac pacemakers who are unable to undergo bioelectrical impedance analysis; (3) Terminal state, receiving palliative care; (4) Relevant case data is missing. This study has been approved by the Ethics Committee of Southwest Hospital, with the ethics number: (B)KY2025235.

### General information collection

Collect the general demographic data (age, gender, education level, etc.), lifestyle (smoking, drinking, etc.) and comorbidities (hypertension, diabetes, blood lipid status, etc.) of the research subjects.

### Diagnosis of sarcopenia

The diagnosis of sarcopenia is based on the 2019 consensus of the Asian Sarcopenia Working Group (10), which includes three aspects: muscle mass, muscle strength and physical function. Muscle mass: The SMI of the extremities was measured by bioelectrical impedance analysis (body composition analyzer InBody 770). Low muscle mass was defined as less than 7.0 kg/m^2^ in males and less than 5.7 kg/m^2^ in females. Muscle strength: The dominant handgrip strength was measured using an electronic handgrip strength meter (EH101, Japan). Low muscle strength was defined as less than 28 kg for men and less than 18 kg for women. Those who meet the criteria of low muscle mass and also have low muscle strength are diagnosed with sarcopenia.

### Cognitive function assessment

Cognitive function was evaluated using the MMSE. The total MMSE score is 30 points, and the cut-off value adjusted for educational attainment is used to diagnose dementia: illiterate ≤ 17 points, primary school ≤ 20 points, secondary school and above ≤ 24 points (11).

### Arteriosclerosis assessment

The baPWV was measured using the Omron arteriosclerosis monitoring device BP-203RPEIII to evaluate the degree of arteriosclerosis. The higher the baPWV value, the more severe the degree of arteriosclerosis it indicates.

### Statistical analysis

Statistical analysis was conducted using SPSS 27.0 software. Measurement data were expressed as mean ± standard deviation (x ± s), and comparisons between groups were conducted using *t*-test or analysis of variance; Counting data were expressed as the number of cases (percentage), and the χ^2^-test was used for comparison between groups. Multiple linear regression analysis was used to explore the relationship among sarcopenia, arteriosclerosis and cognitive function. The Bootstrap method was used for mediating effect analysis. The sample was repeated 1,000 times to calculate the estimated value of the mediating effect and its 95% confidence interval (CI). *P* < 0.05 was considered statistically significant.

## Results

### General information and baseline characteristics

**FIGURE 1:**
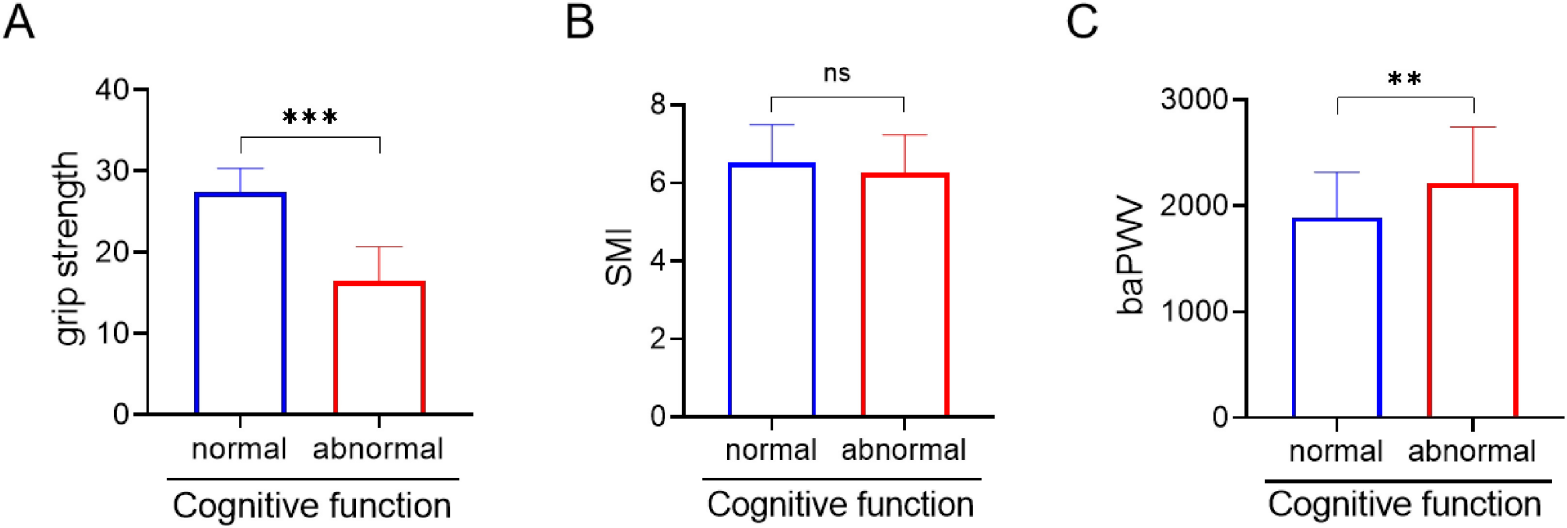
Three bar charts labeled **(A–C)** compare normal and abnormal cognitive function groups. **(A)** Shows significantly higher grip strength for normal group (***P < 0.001). **(B)** Shows no significant difference in SMI (ns:*p* > 0.05). **(C)** Shows baPWV is significantly higher in the abnormal group (**p < 0.01).

Table 1 summarizes the baseline characteristics of the enrolled patients. Among the 99 patients who met the inclusion criteria, 34 were diagnosed with cognitive dysfunction, while 65 had normal cognitive function. Patients with cognitive dysfunction were significantly older than those with normal cognitive function (70.00 [62.00–76.00] vs. 78.50 [72.75–82.25], *P* < 0.001). Compared with the normal cognitive function group, handgrip strength was markedly lower in the cognitive dysfunction group (27.30 [19.90–32.65] vs. 20.65 [16.13–25.00], *P* < 0.001) (Figure 1). In addition, the prevalence of sarcopenia was higher in the cognitive dysfunction group (24.6% vs. 44.1%, *P* = 0.047). However, there was no significant difference in skeletal muscle mass index (SMI) between the two groups (6.60 [5.85–7.20] vs. 6.20 [5.68–6.93], *P* = 0.217) (Figure 1). Notably, brachial–ankle pulse wave velocity (baPWV) was significantly elevated in patients with cognitive dysfunction compared with those with normal cognitive function (1806.00 [1565.00–2178.00] vs. 2232.50 [1818.50–2585.75], *P* = 0.002) (Figure 1).

**TABLE 1:** Baseline characteristics of patients grouped according to whether they have cognitive dysfunction or non-cognitive function.

Characteristic	Total	Non-cognitive function	Cognitive function	*P*-value
Age	73.00[64.00, 80.00]	70.00[62.00, 76.00]	78.50[72.75, 82.25]	<0.001
Gender				0.226
Male	52[52.5]	37[56.9]	15[44.1]	
Female	47[47.5]	28[43.1]	19[55.9]	
Education				0.162
Illiterate	9[9.1]	3[4.6]	6[17.6]	
Primary school	36[36.4]	24[36.9]	12[35.3]	
Junior high school	30[30.3]	21[32.3]	9[26.5]	
High school	11[11.1]	8[12.3]	3[8.8]	
Secondary vocational school	4[4.0]	2[3.1]	2[5.9]	
Junior college	5[5.1]	4[6.2]	1[2.9]	
Undergraduate	4[4.0]	3[4.6]	1[2.9]	
History of smoking				0.184
Yes	38[38.4]	28[43.1]	10[29.4]	
No	61[61.6]	37[56.9]	24[70.6]	
History of drinking				0.993
Yes	35[35.4]	23[35.4]	12[35.3]	
No	64[64.6]	42[64.6]	22[64.7]	
Hypertension				0.298
Yes	63[63.6]	39[60.0]	24[70.6]	
No	36[36.4]	26[40.0]	10[29.4]	
Diabetes				0.077
Yes	27[27.3]	14[21.5]	13[38.2]	
No	72[72.7]	51[78.5]	21[61.8]	
Total cholesterol	4.49[3.74, 5.45]	4.48[3.75, 5.50]	4.49[3.69, 5.5.09]	0.991
Triglyceride	1.39[0.89, 1.85]	1.26[0.88, 1.84]	1.42[0.94, 1.89]	0.441
High-density lipoprotein	1.35[1.11, 1.60]	1.34[1.08, 1.58]	1.35[1.11, 1.66]	0.608
Low-density lipoprotein	2.69[2.07, 3.33]	2.69[2.06, 3.39]	2.69[2.14, 3.13]	0.974
baPWV	1946.00[1622.00, 2285.00]	1806.00[1565.00, 2178.00]	2232.50[1818.50, 2585.75]	0.002
SMI	6.30[5.80, 72.0]	6.60[5.85, 7.20]	6.20[5.68, 6.93]	0.217
Grip strength	23.80[18.80, 29.60]	27.30[19.90, 32.65]	20.65[16.13, 25.00]	<0.001
Sarcopenia				0.047
Yes	31[31.3]	16[24.6]	15[44.1]	
No	68[68.7]	49[75.4]	19[55.9]	

Continuous variables are presented as median (interquartile range, IQR) and were compared using the Mann–Whitney U test. Categorical variables are expressed as counts (percentages) and were compared using the χ^2^-test or Fisher’s exact test, as appropriate.

### The interrelationship among arteriosclerosis, sarcopenia and cognitive dysfunction

**FIGURE 2:**
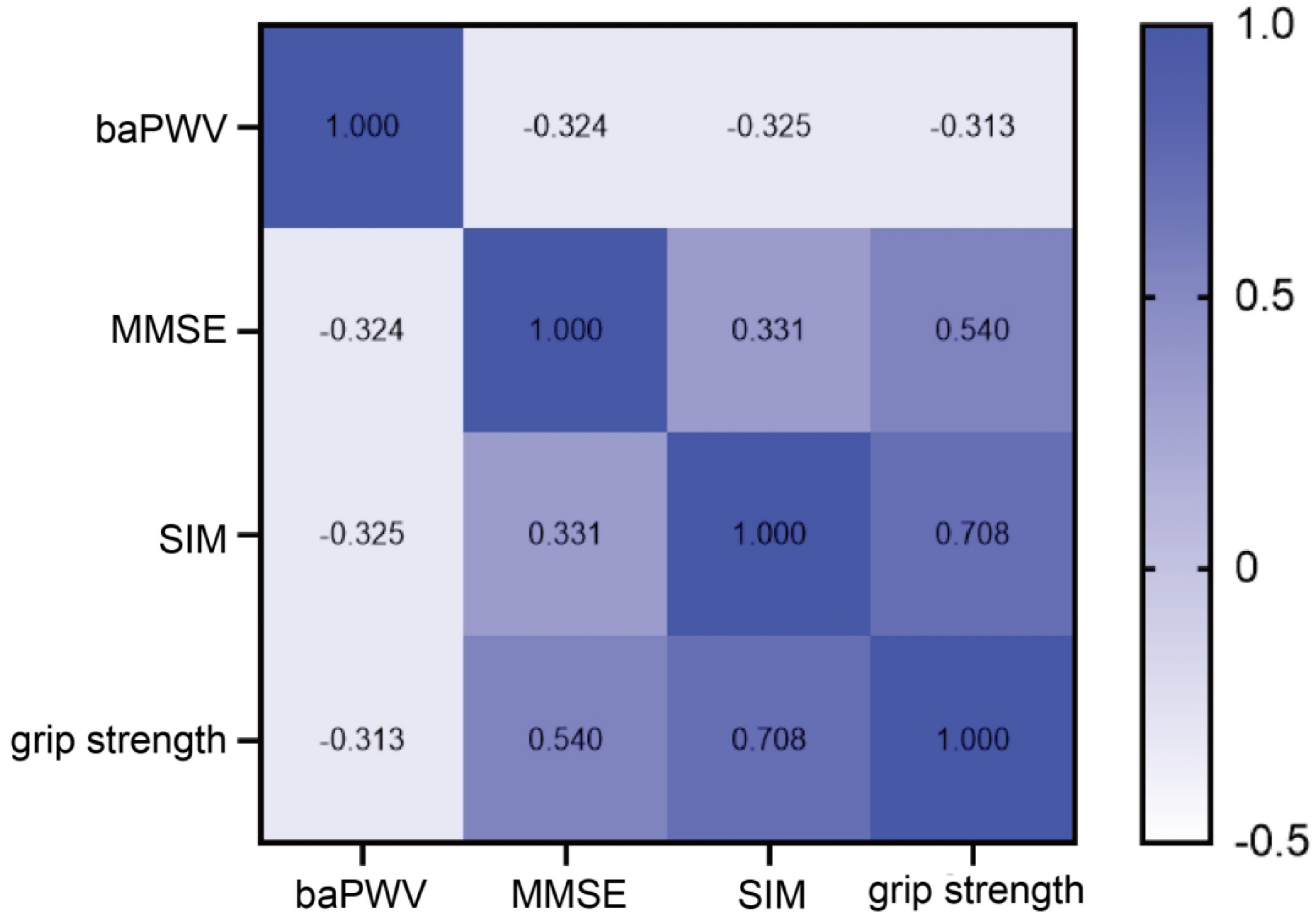
Spearman correlation analysis of the association among baPWV, MMSE, SMI and grip strength. baPWV is negatively correlated with MMSE, SMI and grip strength, while MMSE is positively correlated with SMI and grip strength.

Figure 2 illustrates the associations among baPWV, MMSE, SMI, and grip strength, analyzed using Spearman’s correlation. The results demonstrated that baPWV was negatively correlated with MMSE (*r* = –0.324, *P* = 0.001), SMI (*r* = –0.325, *P* = 0.001), and grip strength (*r* = –0.313, *P* = 0.002). Conversely, MMSE was positively correlated with SMI (*r* = 0.331, *P* = 0.001) and grip strength (*r* = 0.540, *P* < 0.001). To further examine these associations, multiple linear regression analyses were performed. Model 1, without adjustment for confounders, showed that baPWV was negatively associated with MMSE (β = –0.004, *P* < 0.001), SMI (β = –201.239, *P* < 0.001), and handgrip strength (β = –13.732, *P* = 0.025). MMSE was positively associated with SMI (β = 1.267, *P* = 0.040) and grip strength (β = 0.260, *P* < 0.001) (Table 2). Model 2 adjusted for age, smoking status, and diabetes. After adjustment, baPWV remained negatively associated with MMSE (β = –0.003, *P* = 0.047), SMI (β = –194.310, *P* < 0.001), and grip strength (β = –18.541, *P* < 0.001). MMSE remained positively associated with SMI (β = 2.061, *P* = 0.001) and grip strength (β = 0.351, *P* < 0.001) (Table 2). Given that the non–high-density lipoprotein cholesterol to high-density lipoprotein cholesterol ratio (NHHR) is a novel lipid parameter closely linked to increased risk of cognitive dysfunction (12), Model 3 further adjusted for NHHR. After adjustment, baPWV remained negatively correlated with MMSE (β = –0.003, *P* = 0.043), SMI (β = –187.010, *P* < 0.001), and grip strength (β = –14.242, *P* = 0.019). The positive associations between MMSE and SMI (β = 1.361, *P* = 0.040) and grip strength (β = 0.260, *P* < 0.001) also persisted (Table 2).

**TABLE 2:** Multiple linear regression analysis of independent associations among BaPWV, MMSE, SMI, and grip strength (NHHR = non-HDL-C/HDL-C, non-HDL-C = TC-HDL-C).

Independent variable	Dependent variable	Model 1		Model 2		Model 3	
		β(95%CI)	*P*	β(95%CI)	*P*	β(95%CI)	*P*
Grip strength	MMSE	0.351 (0.225–0.478)	<0.001	0.260 (0.116–0.404)	<0.001	0.260 (0.115–0.405)	<0.00
Grip strength	BaPWV	−18.541 (−29.227 to −7.855)	<0.001	−13.732 (−25.672 to −1.792)	0.025	−14.242 (−26.098 to −2.386)	0.019
BaPWV	MMSE	−0.004 (−0.007 to 0.002)	<0.001	−0.003 (−0.005 to −0.000)	0.047	−0.003 (−0.005 to −0.000)	0.043
SMI	BaPWV	−201.239 (−292.483 to −109.995)	<0.001	−194.310 (−288.997 to −99.624)	<0.001	−187.010 (−284.659 to −89.361)	<0.001
SMI	MMSE	2.061 (0.848–3.273)	0.001	1.267 (0.009–2.525)	0.048	1.361 (−0.063 to 2.659)	0.040

Model 1: unadjusted. Model 2: adjusted for age, smoking status, and diabetes. Model 3: Model 2 additionally adjusted for NHHR.

### Mediating effect of arteriosclerosis in grip strength, SMI and MMSE scores

**FIGURE 3:**
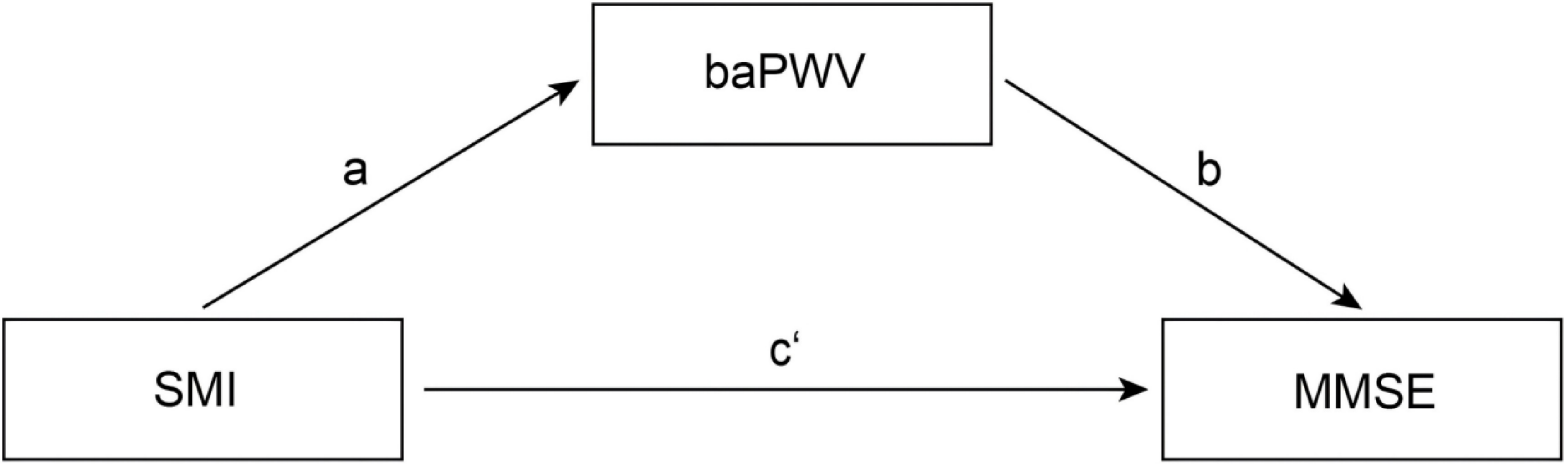
The mediating model relationship of SMI, baPWV and MMSE.

**FIGURE 4:**
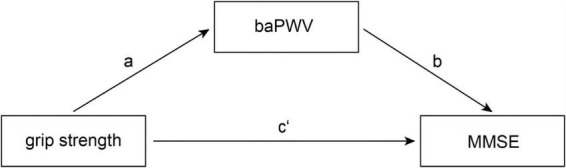
The mediating model relationship of handgrip strength, baPWV and MMSE.

In this study, baPWV was considered as a potential mediating factor to examine whether, and to what extent, it mediates the associations among grip strength, SMI, and MMSE scores (Figures 3, 4). The analyses revealed that grip strength exerted a significant positive effect on MMSE scores (β = 0.3515, *P* < 0.001), with baPWV demonstrating a marginally significant mediating effect (Table 3). Specifically, grip strength indirectly improved cognitive performance by reducing baPWV (β = –18.5410, *P* = 0.001) (Table 2), yielding an indirect effect of ab = 0.0487, 95% CI [–0.0028, 0.1132], which accounted for 13.9% of the total effect (Table 3). Even after accounting for baPWV, the direct effect of grip strength on MMSE remained significant (β = 0.3028, *P* < 0.001), suggesting that its influence may be mediated predominantly through other pathways. After further adjustment for potential confounders including age, diabetes history, smoking status, and NHHR, grip strength continued to show a positive association with MMSE (β = 0.2599, *P* = 0.001) and a negative association with baPWV (β = –14.2423, *P* = 0.019). However, in this model, the indirect effect of baPWV on MMSE was not statistically significant (ab = 0.0239, 95% CI [–0.0192, 0.0759]), indicating that the relationship between grip strength and cognitive function may not be substantially mediated by baPWV (Table 3). Similarly, SMI exhibited a significant positive association with MMSE (β = 2.0607, *P* = 0.001), with baPWV partially mediating this relationship (Table 4). SMI enhanced cognitive function indirectly by lowering baPWV (β = –201.2391, *P* < 0.001) (Table 2), corresponding to an indirect effect of ab = 0.6550, 95% CI [0.0194, 1.3971], and accounting for 31.8% of the total effect (Table 4). After controlling for baPWV, the direct effect of SMI on MMSE remained significant (β = 1.4057, *P* = 0.033), suggesting that SMI may also exert cognitive benefits through alternative biological mechanisms. When additional confounders including age, diabetes history, smoking status, and NHHR were considered, SMI retained a significant overall effect on MMSE (β = 1.3607, *P* = 0.040) and was inversely associated with baPWV (β = –187.0133, *P* < 0.001). However, the indirect effect of baPWV on MMSE was not significant in this fully adjusted model (ab = 0.3573, 95% CI [–0.2534, 1.0031]), indicating that baPWV did not play a consistent mediating role after comprehensive adjustment.

**TABLE 3:** The mediating effect of baPWV in the association between grip strength and MMSE.

Variables	Model 1	Model 2
Total effect	0.3515(0.2247–0.4783)	0.2599(0.1149–0.4048)
Direct effect (c’)	0.3028(0.1710–0.4345)	0.2360(0.0873–0.3847)
Indirect effect (ab)	0.0487(−0.0028 to 0.1132)	0.0239(−0.0192 to 0.0759)
Percent mediation (%)	13.9%	/

Model 1: unadjusted; Model 2: adjusted for age, smoking status, diabetes, and NHHR. Indirect effects were estimated using bootstrap with 1,000 resamples.

**TABLE 4:** The mediating effect of baPWV in the association between SMI and MMSE.

Variables	Model 1	Model 2
Total effect	2.0607(0.8484–3.2730)	1.3607(0.0628–2.6587)
Direct effect (c’)	1.4057(0.1126–2.6988)	1.0034(–0.3850 to 2.3918)
Indirect effect (ab)	0.6550(0.0194–1.3971)	0.3573(−0.2534 to 1.0031)
Percent mediation(%)	31.8%	/

Model 1: unadjusted; Model 2: adjusted for age, smoking status, diabetes, and NHHR. Indirect effects were estimated using bootstrap with 1,000 resamples.

## Discussion

This study aimed to investigate whether arteriosclerosis mediates the relationship between sarcopenia and cognitive dysfunction by evaluating the degree of arteriosclerosis. Sarcopenia was assessed using grip strength and SMI, arteriosclerosis by baPWV, and cognitive function by MMSE. Our preliminary findings revealed that most elderly individuals with cognitive dysfunction also exhibited sarcopenia and greater arterial stiffness. Cognitive impairment is highly prevalent worldwide, affecting approximately 50 million individuals, and the incidence of dementia doubles every 5 years (13–15). Beyond impairing daily living, cognitive dysfunction imposes a substantial burden on healthcare systems and society, given the increased demand for medical and nursing services. Furthermore, cognitive dysfunction often coexists with or accelerates the progression of other conditions. For instance, frailty incidence increases by 19.5% annually among older adults with cognitive impairment (16), and cognitive dysfunction is strongly associated with cardiovascular diseases such as hypertension, coronary artery disease, and atrial fibrillation (17). Sarcopenia, a major component of frailty syndrome, is characterized by age-related declines in skeletal muscle mass and strength, which can lead to disability, reduced quality of life, and mortality (18). Meanwhile, arteriosclerosis has long been established as a critical contributor to cardiovascular disease (19), which remains a leading cause of death among elderly populations.

Despite these established associations, the interplay among arteriosclerosis, sarcopenia, and cognitive dysfunction has not been fully elucidated. In this study, we further explored whether arteriosclerosis functions as a mediator in the link between sarcopenia and cognitive dysfunction. Our results demonstrated that baPWV was negatively correlated with MMSE, SMI, and grip strength, whereas MMSE was positively correlated with SMI and grip strength. Mediation analyses revealed that grip strength had a significant positive association with MMSE scores, with baPWV exerting a marginal mediating effect. Grip strength improved cognitive function partly through reducing baPWV, but this indirect effect accounted for only 13.9% of the total effect, suggesting that the majority of grip strength’s influence on cognitive function may occur via other biological pathways. Notably, after adjustment for age, diabetes, smoking, and NHHR, the mediating role of baPWV was no longer significant. Similarly, SMI showed a significant positive association with MMSE, and baPWV played a partial mediating role, explaining 31.8% of the total effect.

This finding suggests that part of the beneficial effect of skeletal muscle mass on cognitive function may be mediated through vascular pathways, while additional mechanisms are also likely involved. However, after adjusting for the same set of confounders, the mediating role of baPWV was attenuated and no longer significant, indicating that arteriosclerosis may not consistently mediate the sarcopenia–cognition relationship when other cardiovascular and metabolic risk factors are considered. This study highlights a significant association between sarcopenia and cognitive dysfunction, with arteriosclerosis potentially contributing to this relationship. Both grip strength and SMI were positively correlated with cognitive function, although the indirect effects mediated by baPWV differed, with SMI showing a higher proportion. Reduced muscle mass is closely linked to systemic metabolic dysfunction, chronic inflammation, hormonal imbalances, and impaired cerebral perfusion, which may exacerbate cerebral small vessel disease and cognitive decline (20–23). In contrast, grip strength primarily reflects neuromuscular function and represents an integrated outcome of muscle quality and neural regulation. It is more susceptible to influences from the central nervous system, neural conduction efficiency, and short-term functional status (3). Accordingly, the association between grip strength and cognitive function may be mediated more directly through neuromuscular pathways or the “muscle–brain axis,” rather than predominantly through vascular mechanisms. Therefore, it is biologically plausible that SMI accounts for a greater proportion of cognitive decline mediated via arterial stiffness. These findings suggest that vascular-mediated pathways may partly underlie the sarcopenia–cognition relationship. However, after adjusting for confounders including age, diabetes, smoking, and NHHR, the mediating effect of baPWV was no longer significant, indicating that arteriosclerosis may not be the primary pathway. These findings suggest that age, diabetes, and related metabolic factors may occupy a more upstream or central position within the sarcopenia–cognitive impairment pathway. Aging is a well-established common driver of arterial stiffening, skeletal muscle decline, and cognitive deterioration, primarily through mechanisms involving endothelial dysfunction, increased oxidative stress, and mitochondrial impairment (24). In addition, diabetes and insulin resistance not only accelerate the progression of arteriosclerosis but also exert direct neurocognitive effects by disrupting cerebral glucose metabolism, promoting neuroinflammation, and facilitating β-amyloid deposition (25). Accordingly, the inclusion of these variables in the adjusted model likely attenuated the apparent mediating effect of arterial stiffness, suggesting that its role may be better characterized as part of a parallel pathway associated with metabolic dysregulation, rather than as an independent and dominant mediator. Furthermore, NHHR, as an indicator of lipid metabolic imbalance, is closely linked to systemic inflammation, which has been increasingly recognized as a critical biological bridge connecting sarcopenia and cognitive decline (26). Therefore, additional adjustment for metabolic and inflammation-related factors may have further diminished the explanatory contribution of arteriosclerosis in the mediation model.

Peripheral arterial stiffness, reflected by baPWV, may not fully capture cerebral small vessel pathology, which is more closely associated with cognitive impairment (27, 28). Cognitive decline, particularly mild cognitive impairment and vascular dementia is closely linked to cerebral small vessel disease, including white matter hyperintensities, microbleeds, and lacunar infarctions, which have been shown to correlate more strongly with central arterial stiffness, such as cfPWV (29). Furthermore, increased central arterial stiffness can impair the structure and function of the cerebral microcirculation by augmenting pulsatile hemodynamic stress, thereby contributing to the progression of cognitive decline (30). Therefore, the use of baPWV in this study may not have fully captured the critical “central artery–brain microcirculation” pathway, potentially limiting the detection of mediating effects. This also highlights an inherent limitation of the measurement approach, namely its reduced sensitivity in reflecting vascular alterations within specific arterial beds.

In addition to vascular mechanisms, other non-vascular pathways may also mediate the association between sarcopenia and cognitive impairment, including inflammation, oxidative stress, endocrine dysregulation, malnutrition, mitochondrial dysfunction, and physical inactivity (31–35). For example, the emerging concept of the “muscle–brain axis” suggests that skeletal muscle functions as an endocrine organ through the secretion of myokines such as irisin and insulin-like growth factor-1 (IGF-1). These factors may cross the blood-brain barrier (BBB) and exert neuroprotective effects while simultaneously suppressing neuroinflammation (36). Chronic systemic inflammation may also represent an important non-vascular mechanism linking sarcopenia and cognitive impairment. Aging-related increases in pro-inflammatory cytokines, including interleukin-6 (IL-6) and tumor necrosis factor-α (TNF-α), may promote both muscle catabolism and neurodegeneration. Persistent low-grade inflammation can disrupt BBB integrity, activate microglia, and impair synaptic function, thereby accelerating cognitive decline independently of arterial stiffness (37). Therefore, these non-vascular pathways may play a more substantial role in linking sarcopenia to cognitive decline.

Several limitations should be acknowledged. First, baPWV alone may not comprehensively assess arteriosclerosis across different vascular beds; combining carotid IMT or cerebrovascular imaging could improve accuracy. Second, MMSE provides limited sensitivity for detecting mild cognitive impairment, potentially introducing measurement variability. Finally, the retrospective design entails selection bias and precludes causal inference. Prospective, longitudinal studies incorporating biochemical markers, neuroimaging, and multi-mediator models are needed to clarify the dominant pathways and strengthen causal understanding.

From a clinical perspective, our findings highlight the potential importance of integrated, multi-target intervention strategies. Approaches aimed at preserving muscle mass, such as resistance exercise and adequate nutritional support, may be beneficial. Concurrently, optimizing vascular health through the management of blood pressure, glycemic control, and metabolic risk factors may help mitigate arteriosclerosis. These combined strategies may provide synergistic benefits in reducing the risk of cognitive decline, although this hypothesis requires validation in prospective and interventional studies.

## Conclusion

This study demonstrates that sarcopenia is significantly associated with cognitive impairment and increased arteriosclerosis in elderly individuals. Although arteriosclerosis showed a potential mediating effect in unadjusted analyses, this effect was attenuated and became non-significant after full adjustment for confounders, suggesting that its mediating role in the sarcopenia–cognition relationship remains inconclusive. These findings indicate that multiple pathways, rather than a single vascular mechanism, may underlie this association. Future prospective studies incorporating longitudinal designs and multi-mediator models are warranted to further elucidate the underlying mechanisms.

## Data Availability

The raw data supporting the conclusions of this article will be made available by the authors, without undue reservation.
